# PERSPECTIVES ON PERSON-CENTEREDNESS ACROSS HEALTH CARE SETTINGS

**DOI:** 10.1093/geroni/igae098.0677

**Published:** 2024-12-31

**Authors:** Philip Sloane, Sheryl Zimmerman, Ayesha Syed, Sam Fazio

**Affiliations:** University of North Carolina, Chapel Hill, North Carolina, United States; University of North Carolina, Chapel Hill, North Carolina, United States; University of North Carolina, Chapel Hill, North Carolina, United States; Alzheimer’s Association, Chicago, Illinois, United States

## Abstract

Systems perspectives generally identify three levels of action: the interpersonal (micro) level, the institutional (meso) level, and the broader societal (macro) level. Person-centeredness is generally viewed at the interpersonal level, but especially in the context of health and supportive care, broader systems can shape and impact the quality and outcomes of these one-on-one interactions. To better understand how and why person-centeredness varies across health care settings (e.g., hospitals, nursing homes), we conducted interviews and convened think tank meetings with 31 diverse stakeholders including direct care workers, administrative staff, consumer advocates, and academic researchers focusing on care of older persons. Prompts asked examples of person-centeredness (or lack thereof) in numerous settings. The following themes were identified that help explain the wide range in person-centeredness across health care settings: 1) variation in system goals related to person-centeredness; 2) variation in systems’ perceived need to limit person-centered; 3) relationships between resource availability and person-centeredness; 4) cultural values and societal inequity as root causes of much variation in person-centeredness; and 5) influence on person-centeredness of the degree to which hospitality is a core value within a given system. Understanding and responding to these themes within a given setting may more effectively help meet resident needs and preferences; it also has implications for policymaking, such as creation of training guidelines for care staff and benchmarks for program leaders.

